# TDFusion: When Tensor Decomposition Meets Medical Image Fusion in the Nonsubsampled Shearlet Transform Domain

**DOI:** 10.3390/s23146616

**Published:** 2023-07-23

**Authors:** Rui Zhang, Zhongyang Wang, Haoze Sun, Lizhen Deng, Hu Zhu

**Affiliations:** 1 Jiangsu Province Key Lab on Image Processing and Image Communication, Nanjing University of Posts and Telecommunications, Nanjing 210003, China; rayzhangxu@163.com (R.Z.); wangzhongyang96210@163.com (Z.W.); haozesun@163.com (H.S.); 2 National Engineering Research Center of Communication and Network Technology, Nanjing University of Posts and Telecommunications, Nanjing 210003, China; alicedenglzh@gmail.com

**Keywords:** medical image, multimodal fusion, guided filtering, ADMM

## Abstract

In this paper, a unified optimization model for medical image fusion based on tensor decomposition and the non-subsampled shearlet transform (NSST) is proposed. The model is based on the NSST method and the tensor decomposition method to fuse the high-frequency (HF) and low-frequency (LF) parts of two source images to obtain a mixed-frequency fused image. In general, we integrate low-frequency and high-frequency information from the perspective of tensor decomposition (TD) fusion. Due to the structural differences between the high-frequency and low-frequency representations, potential information loss may occur in the fused images. To address this issue, we introduce a joint static and dynamic guidance (JSDG) technique to complement the HF/LF information. To improve the result of the fused images, we combine the alternating direction method of multipliers (ADMM) algorithm with the gradient descent method for parameter optimization. Finally, the fused images are reconstructed by applying the inverse NSST to the fused high-frequency and low-frequency bands. Extensive experiments confirm the superiority of our proposed TDFusion over other comparison methods.

## 1. Introduction

Medical image fusion plays a pivotal role in contemporary medical research and holds profound significance for medical treatments [1]. With the advancements in medical imaging, a diverse array of imaging technologies has emerged. Prominent modalities encompass cardiac angiography, computed tomography (CT), positron emission tomography (PET), magnetic resonance imaging (MRI), and single photon emission computed tomography (SPECT), among others. Each of these modalities focuses on distinct aspects of the human body or various pathologies. Early medical images predominantly captured the anatomical structure through morphological imaging techniques such as CT and MR images. Although both of these modalities provide anatomical information, their emphasis differs. CT effectively reveals bone tissue and blood vessels, while MRI excels in visualizing soft tissue. The advent of PET and SPECT has enhanced the emphasis on functional and metabolic information. Nonetheless, the information conveyed by these individual images remains fragmented, limiting their utility in medical observation and diagnosis. Consequently, medical image fusion technology [2,3], which integrates multiple images of diverse modalities into a single composite image that encompasses a range of complementary information, has achieved increasing attention. This technique mitigates randomness and redundancy, enhancing the clinical applicability of medical images for the diagnosis and assessment of medical conditions.

The prevailing medical image fusion methodologies [4,5,6,7,8,9] primarily adopt a multiscale transformation (MST) framework. In general, MST-based fusion methods entail the following three steps: Firstly, the source images are decomposed into a domain of multiscale transforms. Subsequently, the transformed coefficients are merged utilizing a fusion rule. Finally, the fused image is reconstructed by applying the inverse transform to the merged coefficients. Within this framework, fusion approaches that incorporate diverse transformation methods and fusion strategies have been proposed, including the nonsubsampled contourlet transform (NSCT) [10] and the nonsubsampled shearlet transform (NSST) [11]. While the implementation process of the shearlet transform (ST) method utilized in NSST shares similarities with the contourlet transform (CT), the ST employs shear filters instead of directional filters found in the CT. This distinction liberates ST from the limitations associated with the number of directions, enabling it to capture a greater level of detail and functional information across various orientations. The ST exhibits a heightened directional sensitivity and accommodates diverse geometric shapes, thereby proficiently capturing the intrinsic geometric features of multidimensional phenomena. The NSST incorporates the nonsubsampled ST within nonsubsampled pyramid filters (NSPFs) [12] and the shift-invariant shearlet filters (SFBs) [11]. This integration endows the NSST with an enhanced robustness to distortion, courtesy of the shift variance property. Furthermore, the NSST inherits the advantages of multiscale and multidirectional characteristics, rendering it a highly effective image decomposition method. Given these compelling attributes, the NSST is selected as the preferred multiscale transform for our model. Consequently, our model adheres to the fundamental MST-based fusion framework.

In the context of MST-based fusion methods, the fusion strategy for high-frequency and low-frequency components represents a pivotal challenge. Although the human visual system is more sensitive to high-frequency components, a significant portion of information in the source images is embedded within the low-frequency domain. Thus, achieving meaningful fusion outcomes necessitates careful consideration of both high-frequency and low-frequency fusion processes. Regrettably, existing MST-based fusion methods often address these two problems separately and treat them disparately and independently. The integration of neural networks holds considerable promise for data fusion tasks. Notably, recent works [13,14,15] have leveraged deep neural networks to fuse multimodal information for predicting interactions. Specifically, a fusion strategy based on the fuzzy adaptive reduced pulse-coupled neural network (RPCNN) [16] has demonstrated effective handling of the primary low-frequency and high-frequency fusion problems. By employing RPCNNs with fuzzy-adaptive linking strengths, both the low-frequency subband (LFS) and high-frequency subband (HFS) coefficients are fused in a similar manner. This approach significantly enhances the image fusion performance. However, one limitation remains, which is the presence of free parameters in the pulse-coupled neural network (PCNN). To overcome the challenge of setting free parameters in the traditional PCNN model, Ming et al. [7] introduced the parameter adaptive pulse-coupled neural network (PA-PCNN) model to fuse high-frequency coefficients using adaptive PCNN parameters. While this method addresses the parameter selection issue, the fusion strategy for the high-frequency component remains conventional, adhering to the traditional high-frequency fusion approach. Xu et al. [17] exploited the component separation setting, but its complex computation degrades its practicability. In order to equally emphasize the high- and low-frequency components, we propose a cross-fusion technique that integrates the high-frequency and low-frequency components from the source images. In our model, we employ tensor decomposition to effectively fuse the high-frequency and low-frequency components, thereby extracting more pertinent information.

Tensors, as a branch of mathematical research and a generalization of vector concepts, offer great convenience in handling high-dimensional data. With the advancements in computational imaging like hyperspectral imaging and magnetic resonance imaging, tensors have gradually found practical applications. Tensor decomposition serves as a higher-order generalization of matrix decomposition, sharing similarities with matrix factorization. It serves three main purposes: dimension reduction, missing data filling, and implicit relation mining. In the context of multi-dimensional images, the Tucker decomposition [18] has been extensively utilized for various purposes, such as image denoising, selecting image features through tensor subspace, and compressing data. Building upon Tucker decomposition, Li et al. proposed a multiband image fusion method based on tensor decomposition [19]. This approach approximates the three-dimensional tensor as a nuclear tensor multiplied by a three-order dictionary, expressing the fusion problem as an estimation of the kernel tensor and the three-mode dictionary. Through iterative decomposition steps until convergence, the dictionary and core tensor are updated to achieve accurate estimation. In our proposed method, we construct an image fusion strategy based on tensor decomposition, leveraging the fusion of tensor information from different dimensions (with high frequency being three-dimensional and low frequency being two-dimensional). By cross-fusing the high- and low-frequency components in the source images, we aim to achieve equal attention to the high- and low-frequency components. In our paper, we combine the tensor-decompositon-based fusion strategy with the nonsubsampled shearlet transform (NSST) to develop a novel optimization model for medical image fusion, named tensor decomposition in the nonsubsampled shearlet transform domain (TDFusion).

In the field of medical image fusion, the concept of tensors has been previously explored but has not been considered a sufficiently novel idea. For instance, Liu et al. [20] proposed the use of structure tensors for analyzing image properties. However, it is important to note that the structure tensor differs fundamentally from the tensor employed in our model. Our tensor representation serves as a method to effectively represent image data and facilitates improved fusion outcomes. During the Tucker decomposition process, there is a potential risk of damaging certain information in the source material due to the sparse prior. To address this issue, we introduce the joint static and dynamic guidance filtering (JSDG) technique proposed by Ham et al. [21] to supplement the corresponding high-frequency information. Unlike the multi-modal deep guided filtering approach proposed by Bernhard et al. [22], which combines a local linear guided filter with a guided image obtained from multimodal input, JSDG incorporates the concept of dynamic and static guidance and utilizes the output as the dynamic guidance of the image. This approach not only focuses on the structural information of the static guidance but also considers the properties of the input image. The JSDG model we employ combines static and dynamic guidance structures, effectively incorporating high-frequency components while preserving the results of mixed-frequency fusion. Furthermore, we adopt the low-frequency information completion strategy from the PA-PCNN method, which defines two new activity level measures, namely weighted local energy (WLE) and the weighted sum of an eight-neighborhood-based modified Laplacian (WSEML). In this method, the fusion of low-frequency components from two images is accomplished through the utilization of WLE and WSEML as the foundation of the NSST. This successfully completes the fusion of low-frequency images and information, thereby enhancing the overall fusion performance.

**Figure 1:**
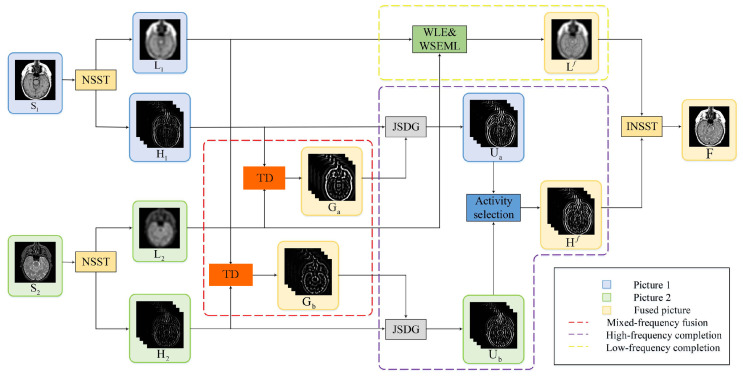
This is the image fusion process of the TDFusion model. Firstly, the source images $S_{1}$ and $S_{2}$ are decomposed into low-frequency $L_{1}$ and $L_{2}$ and high-frequency $H_{1}$ and $H_{2}$ by the NSST method. Then, high-frequency $H_{1}$ and low-frequency $L_{2}$ are fused by the tensor decomposition method to obtain $G_{a}$, and low-frequency $L_{1}$ and high-frequency $H_{2}$ are fused by the same method to obtain $G_{b}$. The obtained $G_{a}$ and $G_{b}$ are added to the JSDG guided filter and the corresponding information from high-frequency $H_{1}$ and $H_{2}$ is added, while the WLE and WSEML methods are used to complete the low-frequency part information. Finally, the fused image is reconstructed by performing the inverse NSST on the fused high-frequency $H_{f}$ and low-frequency $L_{f}$.

In conclusion, this paper proposes a novel unified optimization model for multimodal medical image fusion, leveraging tensor decomposition and the NSST. By integrating the low-frequency and high-frequency components using the tensor decomposition method, we obtain a mixed-frequency fusion image as illustrated in Figure 1. The main contributions of our method can be summarized as follows:

- Our TDFusion model is a unified optimization model. On the basis of the NSST method and the tensor decomposition method, the mixed-frequency fusion image is obtained by fusing the high-frequency and low-frequency components of two source images.
- Considering the structural differences between high-frequency and low-frequency components, some information will be lost during fusion. We embed the framework into the guided filter to optimize and complete the knowledge from low frequencies to high frequencies.
- We combine the ADMM algorithm with the gradient descent method to improve the performance of the fusion image. Through a large number of experiments, the effectiveness of our model in five benchmark datasets of image fusion problems (T1 and T2, T2 and PD, CT and MRI, MRI and PET, and MR and SPECT) is verified. Compared with the other five medical image fusion methods, our model also achieves better results.

The paper structure is arranged as follows. Section 1 is the introduction of the paper and details some methods used in our model. In Section 3, the notation and preliminaries of tensors are briefly introduced. Section 4 discusses the solution process of our model in detail. Then, the specific experimental results and a comparative analysis are shown in Section 5. Finally, Section 6 gives a brief summary.

## 2. Related Work

In recent years, many well-known approaches based on machine learning, deep learning, or some other methods for brain tumor detection and identification have emerged. Diwakar et al. [23] used the non-subsampling shearlet transform (NSST) to extract low- and high-frequency image components in multimodal medical images and proposed a new method for low frequency component fusion based on an improved and modified Laplacian (MSML) clustering dictionary learning technique. In the NSST domain, directional contrast can be used to fuse high-frequency coefficients while using the inverse NSST method to obtain multimodal medical images. Two-dimensional medical image segmentation models are popular among researchers using traditional and new machine learning and deep learning techniques. However, because so much work has been done in recent years to create 3D volumes, 3D volumetric data have just become more widely available. The new architecture developed by Nodirov et al. [24] is based on a 3D U-Net model that employs numerous skip connections, cost-effective pre-trained 3D MobileNetV2 blocks, and attention modules. They employ 3D brain image data. In order to maximize the use of features, additional skip connections are also introduced between the encoder and decoder blocks to streamline the exchange of extracted features between the two blocks. The skip connection’s irrelevant aspects are also filtered out using the attention module, which keeps more processing power while achieving improved accuracy. Biomedical image processing makes it simpler to find and localize brain cancers using MRI. In order to detect brain tumor locations, Arif et al. [25] suggested a method using MRI sequence pictures as the input images. This method is extremely challenging due to the huge range of tumor tissues present in different patients. To enhance the effectiveness of medical image segmentation and streamline the segmentation process, researchers used the Berkeley wavelet transform (BWT) and a deep learning classifier as the foundation for their work. Using the gray-level co-occurrence matrix (GLCM) approach, the important features of each tissue were also identified and the features were subsequently improved using genetic algorithms.

## 3. Notation and Preliminaries of Tensors

We summarize the notation and preliminaries of tensors widely used in this paper in Table 1. The scalars, vectors, matrices, and tensors are, respectively, denoted by the lowercase letters, bold lowercase letters, bold uppercase letters, and calligraphic letters. E means the identity matrix. 1 denotes a matrix filled with ones. Variables with the superscript pre mean the corresponding variables in the previous iteration. The superscript T denotes the transposition operation.

An *M*-dimensional tensor is denoted as $A\in R^{N_{1}\times N_{2}\times \ldots \times N_{M}}$. Its entries are denoted as $a_{n_{1}n_{2},\ldots ,n_{M}}$ or $A\left(n_{1},n_{2},\ldots ,n_{M}\right)$, where $\left(1\leq n_{m}\leq N_{m}\right)$. The *m*-mode unfolding matrix $A_{\left(m\right)}\in R^{N_{m}\times N_{1}N_{2},\ldots ,N_{m-1}N_{m+1},\ldots ,N_{M}}$ is defined by arranging all the *m*-mode unfolding vectors as the rows of the matrix.

Based on the definitions above, we also provide the definition for the multiplication of a tensor and a matrix. Given an *M*-dimensional tensor $A\in R^{N_{1}\times N_{2}\times \ldots \times N_{M}}$ and a matrix $B\in R^{E_{m}\times N_{m}}$, the *m*-mode product can be denoted by $A\times_{m}B$. We record it as $C\in R^{N_{1}\times \ldots \times N_{m-1}\times E_{m}\times N_{m+1}\times \ldots \times N_{M}}$, which is an *M*-dimensional tensor whose entries are computed by:

(1) $$\begin{array}{c} c_{n_{1}n_{2},\ldots ,n_{m-1},e_{m},n_{m+1},\ldots ,n_{M}}=\sum_{n_{m}=1}^{N_{m}}a_{n_{1}n_{2},\ldots ,n_{m-1},n_{m},n_{m+1},\ldots ,n_{M}}b_{e_{m},n_{m}} \end{array}$$

Utilizing the *m*-mode unfolding matrix, the *m*-mode product can also be computed in the form of matrix multiplication:

(2) $$\begin{array}{c} C_{\left(m\right)}={\mathrm{BA}}_{\left(m\right)}=\mathrm{error}\left(A\times_{m}B\right) \end{array}$$

The *m*-mode product also has many properties. We list some of them which will be used in the following deduction.

**Property** **1.** *A series of tensor products are exchangeable for distinct modes (matrices $B_{m_{1}}\in R^{E_{m_{1}}\times N_{m_{1}}},B_{m_{2}}\in R^{E_{m_{2}}\times E_{m_{2}}}$):*

(3) $$\begin{array}{c} A\times_{m_{1}}B_{m_{1}}\times_{m_{2}}B_{m_{2}}=A\times_{m_{2}}B_{m_{2}}\times_{m_{1}}B_{m_{1}}\left(m_{1}\neq m_{2}\right) \end{array}$$

**Property** **2.** *A series of tensor products are mergeable for the same mode (matrices $B_{1}\in R^{E_{m}\times N_{m}},$$B_{2}\in R^{L_{m}\times E_{m}}$):*

(4) $$\begin{array}{c} A\times_{m}B_{1}\times_{m}B_{2}=A\times_{m}\left(B_{2}B_{1}\right) \end{array}$$

**Property** **3.** *Given a collection of matrices $B_{m}\in R^{E_{m}\times N_{m}}$ (m=1,2,…,M), we define an M-dimensional tensor $G=A\times_{1}B_{1}\times_{2}B_{2}\times_{3}\ldots \times_{M}B_{M}$. Then, for tensor $G\in R^{E_{1}\times E_{2}\times \ldots \times E_{M}}$, we have:*

(5) $$\begin{array}{c} g=\left(B_{M}⊗B_{M-1}⊗\ldots ⊗B_{1}\right)a \end{array}$$

*where* ⊗ *means the Kronecker product and g,a are the vectorization of tensors G,A, which are obtained by stacking all the one-mode vectors of the tensors.*

The symbols used in this section like A,B,C,G represent general tensors or matrices.

## 4. The Proposed Method

After Section 3 introduced the related theorems and other knowledge, in this section, we introduce the NSST, tensor decomposition, and other related content and list the optimization process and the solution process of subproblems.

### 4.1. Nonsubsampled Shearlet Transform (NSST)

A shearlet is able to capture the intrinsic geometrical features of multidimensional phenomena effectively [11]. Owing to the shift invariance given by the nonsubsampled process, the NSST is more robust than other multiscale transforms. As a result, we adopt the NSST in our MST framework, which involves two basic steps:

Multiscale Decomposition: Given the *n*-th source picture $S_{n}\in R^{I\times J}$ in our fusion method, nonsubsampled pyramid filters (NSPFs) are used to obtain multiscale representations of the picture. In total, *K* levels of NSPFs are used, so the source image $S_{n}$ is decomposed into *K* high-frequency maps $H_{n}^{k}\in R^{I\times J}\left(k=1,\ldots ,K\right)$, whose scale ranges from fine to coarse. The rest of picture after filtering is denoted as a low-frequency map $L_{n}\in R^{I\times J}$.

Multidirectional Representation: We let the high-frequency map $H_{n}^{k}$ pass through the shift-invariant shearlet filter banks (SFBs) to obtain its multidirectional representations. If we decompose the *k*-th level of the map in $D_{k}$ directions, the result can be denoted as $H_{n}^{k,d_{k}}\in R^{I\times J}\left(d_{k}=1,\ldots ,D_{k}\right)$.

After the two steps, we obtain the multiscale and multidirectional representations of the source image $S_{n}$: $H_{n}^{k,d_{k}}$, $L_{n}\left(k=1,\ldots ,K,d_{k}=1,\ldots ,D_{k}\right)$. It is worth noting that every decomposed high-frequency map $H_{n}^{k,d_{k}}\in R^{I\times J}$ is of the same size as the source image $S_{n}\in R^{I\times J}$. Thus, we can combine all maps along the third dimension and form a three-dimensional tensor $H_{n}\in R^{I\times J\times D}$, where $D=\sum_{k}d_{k}$. The low-frequency map $L_{n}$ can also be seen as a two-dimensional tensor $L_{n}$. Then, the whole NSST can be represented by the following equation:

(6) $$\begin{array}{c} \mathrm{NSST}\left(S_{n}\right)=\left\{H_{n},L_{n}\right\} \end{array}$$

**Figure 2:**
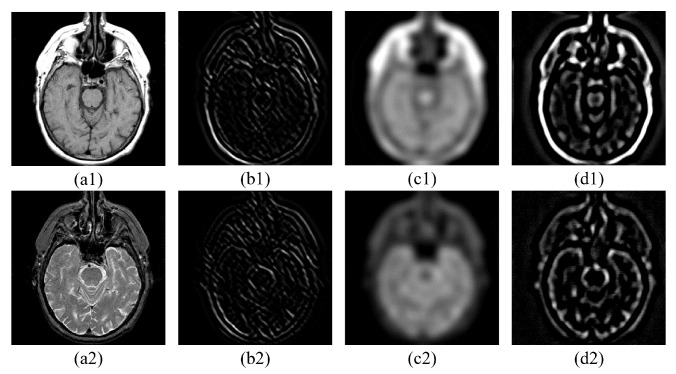
This figure displays the intermediate results of the fusion process. (**a1**,**a2**) are the source images; (**b1**,**b2**) are slices of the high-frequency maps $H_{1},H_{2}$; (**c1**,**c2**) are the low-frequency maps $L_{1},L_{2}$; (**d1**,**d2**) are the slices of the mixed-frequency maps.

For simplicity, we call $H_{n}\in R^{I\times J\times D}$ and $L_{n}\in R^{I\times J}$ the high-frequency and low-frequency maps, respectively, in the following deduction. As can be seen in Figure 2, the high-frequency maps (Figure 2(b1,b2)) contain rich detail and edge information. Human eyes are also more sensitive to the high-frequency information. The low-frequency maps (Figure 2(c1,c2)) mainly preserve the shape and strength information. They keep the majority of information from the source images. Thus, both the high-frequency and low-frequency fusion processes are of great importance to the purpose of medical image fusion, preserving all valid and useful pattern information from the source images.

### 4.2. Tensor Decomposition Based Fusion

In contrast to conventional MST-based fusion strategies, which typically handle high-frequency and low-frequency components individually, we employ a mixed-frequency fusion of the outcomes of the NSST. Specifically, given *N* source images $S_{n}\left(n=1,\ldots ,N\right)$, we perform cross-fusion on their high-frequency maps $H_{n}\left(n=1,\ldots ,N\right)$ and low-frequency maps $L_{n}\left(n=1,\ldots ,N\right)$. The rule of cross-fusion is that a high-frequency map should not be fused with the low-frequency map from the same image. For instance, in the case of two source images, the fusion strategy is $H_{1}\&L_{2}$, $H_{2}\&L_{1}$. Mixed-frequency fusion breaks down the information barrier between the low-frequency and high-frequency components in traditional MST-based strategies and promotes a better fusion of different multiscale information. It also means our model gives equal attention to the high-frequency and low-frequency representations, which are often unequally treated in most medical image fusion methods. Noting that $H_{n}\in R^{I\times J\times D}$ and $L_{n}\in R^{I\times J}$ are tensors of different dimensions, we adopt the tensor-decomposition-based method in [19] to perform our mixed-frequency fusion. Denoting the mixed-frequency maps as $G_{a,b}\in R^{I\times J\times D}$, they can be modeled as a core tensor multiplied by the factor matrix along each mode using the Tucker decomposition [18]:

(7) $$\begin{array}{c} G_{a}=\mathrm{TD}\left(H_{1},L_{2}\right)=C_{a}\times_{1}I_{a}\times_{2}J_{a}\times_{3}D_{a} \end{array}$$

(8) $$\begin{array}{c} G_{b}=\mathrm{TD}\left(H_{2},L_{1}\right)=C_{b}\times_{1}I_{b}\times_{2}J_{b}\times_{3}D_{b} \end{array}$$

where $I_{a,b}\in R^{I\times N_{i}}\left(N_{i}<I\right),J_{a,b}\in R^{J\times N_{j}}\left(N_{j}<J\right)$, and $D_{a,b}\in R^{D\times N_{d}}\left(N_{d}<D\right)$ are dictionaries of the *I*-mode, *J*-mode, and *D*-mode, respectively. Tensors $C_{a,b}\in R^{N_{i}\times N_{j}\times N_{d}}$ hold the coefficients over the three dictionaries. It is noteworthy that although we exclusively discuss the fusion of two images in this context (which is the most prevalent scenario), it is straightforward to extend the model to encompass the fusion of multiple images. From Equations (7) and (8), we can see that they share the same iteration process. For simplification, we delete the subscript to show the generality. Thus, we present our proposed model by the following equation:

(9) $$\begin{array}{c} G=\mathrm{TD}\left(H,L\right)=C\times_{1}I\times_{2}J\times_{3}D \end{array}$$

According to Property 3 in Section 3, the cost function for the mixed-frequency fusion problem F(I,J,D,C) can be written as:

(10) $$\begin{array}{cc} \underset{I,J,D,C}{\mathrm{argmin}} & \;\;\;\;\;{∥H-C\times_{1}I\times_{2}J\times_{3}D∥}_{F}^{2}+\gamma {∥L-C\times_{1}I\times_{2}J\times_{3}D^{*}∥}_{F}^{2}+\lambda {∥C∥}_{1}\\ \; & s.t.D^{*}=PD \end{array}$$

where γ is the fusion control parameter, and λ is the sparsity regularization parameter. The high-frequency map $H\in R^{I\times J\times D}$ is the same size as the mixed-frequency map $G\in R^{I\times J\times D}$. The low-frequency map $L\in R^{I\times J}$ can be viewed as the down-sampled version of the mixed-frequency map G along the third dimension. $P\in R^{1\times D}$ can be understood as the proportion of low-frequency information each direction contains in the mixed-frequency map. In order to solve problem (10), we use the proximal alternating optimization (PAO) algorithm [26], which is guaranteed to converge to a critical point under specific circumstances.

(11) $$\begin{array}{c} \left\{\begin{array}{c} I=\underset{I}{\mathrm{argmin}}F\left(I,J,D,C\right)+\beta {∥I-I^{pre}∥}_{F}^{2}\\ J=\underset{J}{\mathrm{argmin}}F\left(I,J,D,C\right)+\beta {∥J-J^{pre}∥}_{F}^{2}\\ D=\underset{D}{\mathrm{argmin}}F\left(I,J,D,C\right)+\beta {∥D-D^{pre}∥}_{F}^{2}\\ C=\underset{C}{\mathrm{argmin}}F\left(I,J,D,C\right)+\beta {∥C-C^{pre}∥}_{F}^{2} \end{array}\right. \end{array}$$

where F(I,J,D,C) is the objective function in problem (10); β is a positive model parameter; and variables with the superscript pre mean the corresponding variables in the previous iteration. ${∥\cdot ∥}_{F}$ denotes the Forbenius norm. We will provide the solving process of the four subproblems briefly.

### 4.3. The Optimization Solution

#### 4.3.1. Solution of I

Substituting function *F* into the first subequation in Equation (11) and discarding the terms irrelevant to the optimization objective, we obtain the following Equation (12):

(12) $$\begin{array}{c} \underset{I}{\mathrm{argmin}}{∥H-C\times_{1}I\times_{2}J\times_{3}D∥}_{F}^{2}+\gamma {∥L-C\times_{1}I\times_{2}J\times_{3}D^{*}∥}_{F}^{2}+\beta {∥I-I^{pre}∥}_{F}^{2} \end{array}$$

Utilizing the one-mode unfolding matrix and Property 1 of the tensor product in Section 3, we can write Equation (12) in an equivalent form:

(13) $$\begin{array}{c} \underset{I}{\mathrm{argmin}}{∥H_{\left(1\right)}-IA_{i}∥}_{F}^{2}+\gamma {∥L_{\left(1\right)}-IB_{i}∥}_{F}^{2}+\beta {∥I-I^{pre}∥}_{F}^{2} \end{array}$$

where $A_{i}={\left(C\times_{2}J\times_{3}D\right)}_{\left(1\right)},B_{i}={\left(C\times_{2}J\times_{3}D^{*}\right)}_{(1)\cdot }{\left(\cdot \right)}_{\left(1\right)}$ denotes the one-mode unfolding matrix. Equation (13) is quadratic and we can solve the derivative of it:

(14) $$\begin{array}{c} I\left(A_{i}A_{i}^{T}+\gamma B_{i}B_{i}^{T}+\beta E\right)=H_{\left(1\right)}A_{i}^{T}+\gamma L_{\left(1\right)}B_{i}^{T}+\beta I^{pre} \end{array}$$

According to [19], the unique solution of Equation (14) can be efficiently found by the conjugate gradient (CG) algorithm [26].

#### 4.3.2. Solution of J

Substituting function *F* into the second subequation of (11), we obtain problem (15):

(15) $$\begin{array}{c} \underset{J}{\mathrm{argmin}}{∥H-C\times_{1}I\times_{2}J\times_{3}D∥}_{F}^{2}+\gamma {∥L-C\times_{1}I\times_{2}J\times_{3}D^{*}∥}_{F}^{2}+\beta {∥J-J^{pre}∥}_{F}^{2} \end{array}$$

Utilizing the two-mode unfolding matrix and Property 1 of the tensor product in Section 3, we can write Equation (15) in an equivalent form:

(16) $$\begin{array}{c} \underset{J}{\mathrm{argmin}}{∥H_{\left(2\right)}-JA_{j}∥}_{F}^{2}+\gamma {∥L_{\left(2\right)}-JB_{j}∥}_{F}^{2}+\beta {∥J-J^{pre}∥}_{F}^{2} \end{array}$$

where $A_{j}={\left(C\times_{1}I\times_{3}D\right)}_{\left(2\right)}$, $B_{j}={\left(C\times_{1}I\times_{3}D^{*}\right)}_{(2)\cdot }$${\left(\cdot \right)}_{\left(2\right)}$ denotes the two-mode unfolding matrix. Problem (16) is quadratic and we can solve the derivative of it:

(17) $$\begin{array}{c} J\left(A_{j}A_{j}^{T}+B_{j}B_{j}^{T}+\beta E\right)=H_{\left(2\right)}A_{j}^{T}+\gamma L_{\left(2\right)}B_{j}^{T}+\beta J^{pre} \end{array}$$

Considerting to the solving process of I, the unique solution of Equation (17) can be efficiently found by the conjugate gradient (CG) algorithm.

#### 4.3.3. Solution of D

Substituting function *F* into the third subequation of Equation (11), we obtain Equation (18):

(18) $$\begin{array}{c} \underset{D}{\mathrm{argmin}}{∥H-C\times_{1}I\times_{2}J\times_{3}D∥}_{F}^{2}+\gamma {∥L-C\times_{1}I\times_{2}J\times_{3}D^{*}∥}_{F}^{2}+\beta {∥D-D^{pre}∥}_{F}^{2} \end{array}$$

Utilizing the three-mode unfolding matrix in Section 3, we can write Equation (18) in an equivalent form:

(19) $$\begin{array}{c} \;\underset{D}{\mathrm{argmin}}\;{∥\;H_{\left(3\right)}\;-\;DA_{d}\;∥}_{F}^{2}+\gamma {∥\;L_{\left(3\right)}\;-\;\mathrm{PD}A_{d}\;∥}_{F}^{2}+\beta {∥D-D^{pre}∥}_{F}^{2} \end{array}$$

where $A_{d}={\left(C\times_{1}I\times_{2}J\right)}_{(3)\cdot }{\left(\cdot \right)}_{\left(3\right)}$ denotes the three-mode unfolding matrix. Equation (19) is quadratic and we can solve the derivative of it:

(20) $$\begin{array}{c} D\left(A_{d}A_{d}^{T}+\beta E\right)+\gamma P^{T}\mathrm{PD}A_{d}A_{d}^{T}\\ =H_{\left(3\right)}A_{d}^{T}+\gamma P^{T}L_{\left(3\right)}A_{d}^{T}+\beta D^{pre} \end{array}$$

The unique solution of Equation (20) can also be efficiently found by the conjugate gradient (CG) algorithm.

#### 4.3.4. Solution of C

Substituting function *F* into the fourth subproblem in Equation (11), we obtain Equation (21):

(21) $$\begin{array}{c} \underset{C}{\mathrm{argmin}}{∥H-C\times_{1}I\times_{2}J\times_{3}D∥}_{F}^{2}+\gamma {∥L-C\times_{1}I\times_{2}J\times_{3}D^{*}∥}_{F}^{2}+\lambda {∥C∥}_{1}+\beta {∥C-C^{pre}∥}_{F}^{2} \end{array}$$

By introducing the splitting variables $C_{1}=C_{2}=C\in R^{N_{i}\times N_{j}\times N_{d}}$, the problem above can be rewritten as follows:

(22) $$\begin{array}{cc} \underset{C,C_{1},C_{2}}{\mathrm{argmin}} & G\left(C\right)+G_{1}\left(C_{1}\right)+G_{2}\left(C_{2}\right)\\ \; & \;s.t.\;C_{1}=C_{2}=C \end{array}$$

where

(23) $$\begin{array}{c} \left\{\begin{array}{c} G\left(C\right)=\lambda {∥C∥}_{1}+\beta {∥C-C^{pre}∥}_{F}^{2}\\ G_{1}\left(C_{1}\right)={∥H-C\times_{1}I\times_{2}J\times_{3}D∥}_{F}^{2}\\ G_{2}\left(C_{2}\right)=\gamma {∥L-C\times_{1}I\times_{2}J\times_{3}D^{*}∥}_{F}^{2} \end{array}\right. \end{array}$$

Equation (22) can be solved by the alternating direction method of multipliers (ADMM) algorithm [27], the details of which are described in [19]. The optimized $I_{a,b},J_{a,b},D_{a,b},C_{a,b}$ can be obtained after the execution of the PAO algorithm (the convergence of which will be verified in Section 4). Then, we can count the mixed-frequency maps $G_{a},G_{b}$ using Equations (7) and (8). As can be seen in Figure 2(d1,d2), the mixed-frequency maps retain the texture information of high-frequency maps as the basics and simultaneously introduce the features of low-frequency maps. However, it is inevitable that information will be lost in the process of tensor optimization [28]. Thus, we use joint static and dynamic guidance (JSDG) and WLE and WSEML low-frequency fusion to complete the information and promote a better fusion of knowledge. We verify the importance of this process in the ablation analysis.

### 4.4. High-Frequency Completion

#### 4.4.1. Joint Static and Dynamic Guidance

We use joint static and dynamic guidance (JSDG) to complete the high-frequency information in mixed-frequency maps $G_{a},G_{b}$. JSDG is able to jointly leverage the structural information of guidance and input images, so we achieve our purpose of preserving the results of mixed-frequency fusion and perform high-frequency completion at the same time. We also demonstrate its advantages over ordinary guided filtering in the ablation analysis. Denoting the completed mixed-frequency maps as $U_{a,b}\in R^{I\times J\times D}$, the problem can be written as:

(24) $$\begin{array}{c} U_{a}=\mathrm{JSDG}\left(H_{1},G_{a}\right) \end{array}$$

(25) $$\begin{array}{c} U_{b}=\mathrm{JSDG}\left(H_{2},G_{b}\right) \end{array}$$

where the mixed-frequency maps $G_{a,b}$ serve as the static guidance. The high-frequency maps $H_{1,2}$ serve as the input image and dynamic guidance. Since Equations (24) and (25) share the same solving process, we delete the subscript to show the generality. Thus, the purpose of our model is studying the following problem:

(26) $$\begin{array}{c} U=\mathrm{JSDG}(H,G) \end{array}$$

Since all variables in the problem above are three-dimensional tensors, we extend JSDG from 2D signals in [21] to 3D signals. Utilizing the definition of an *m*-mode unfolding vector in Section 2, the cost function of Equation (26) can be denoted as:

(27) $$\begin{array}{c} \underset{U}{\mathrm{argmin}}\;\;\;E\left(U\right)=\sum_{d}{∥u_{d}-h_{d}∥}_{F}^{2}+\alpha \sum_{1\leq m,n\leq IJ}\phi_{\delta }\left[g_{d}\left(m\right)-g_{d}\left(n\right)\right]\psi_{v}\left[u_{d}\left(m\right)-u_{d}\left(n\right)\right] \end{array}$$

where $\phi_{\delta }\left(x\right)=e^{-\delta x^{2}}$, $\psi_{v}\left(x\right)=\left(1-\phi_{v}\left(x\right)\right)/v$,$\psi_{v}\left(x\right)$ is Welsch’s function, which is nonconvex. α is a regularization parameter and δ,v are parameters that control the smoothness bandwidth. $u_{d}\in R^{1\times IJ}\left(d=1\ldots D\right)$ is the three-mode unfolding vector of tensor U, and $h_{d},g_{d}$ have the same meaning. Without loss of generality, we give the same confidence to every pixel. The problem can also be written in an equivalent form:

(28) $$\underset{U}{\mathrm{argmin}}E(U)=1_{D}\left(U_{\left(3\right)}-H_{\left(3\right)}\right){\left(U_{\left(3\right)}-H_{\left(3\right)}\right)}^{T}1_{D}^{T}+\frac{\alpha }{v}1_{IJ}\sum_{d}w_{d}^{g}-w_{d}^{g}w_{d}^{u}1_{IJ}^{T}$$

where $w_{g}^{d}\left(m,n\right)\in R^{IJ\times IJ}=\phi_{\delta }\left(g_{d}\left(m\right)-g_{d}\left(n\right)\right)$, $w_{d}^{u}\left(m,n\right)\in R^{IJ\times IJ}=\phi_{v}\left(u_{d}\left(m\right)-u_{d}\left(n\right)\right)\left(m,n=1,\ldots ,IJ\right)$. ⊗ represents the Hadamard product of the matrices. $1_{x}\in R^{1\times x}$ denotes a vector filled with ones. Equation (28) is a nonconvex optimization problem, which can be solved by the majorization-minimization algorithm presented in [21]. Firstly, we built the surrogate objective function Q(U):

(29a) $$Q\left(U\right)=1_{D}\left(\delta U_{\left(3\right)}Z^{pre}U_{\left(3\right)}^{T}-2H_{\left(3\right)}U_{\left(3\right)}^{T}+H_{\left(3\right)}H_{\left(3\right)}^{T}\right.\left.-\delta U_{\left(3\right)}^{pre}Z^{pre}U_{\left(3\right)}^{preT}\right)1_{D}^{T}+\frac{\alpha }{v}1_{IJ}\Omega^{pre}1_{IJ}^{T}$$

(29b) $$Z=\sum_{d}O^{d}-{w^{g}}_{d}⊗{w^{u}}_{d}$$

(29c) $$O^{d}=\mathrm{diag}\left(o_{1}^{d},\ldots ,o_{IJ}^{d}\right)$$

(29d) $$o_{m}^{d}=\sum_{n=1}^{IJ}\phi_{\delta }\left[g_{d}\left(m\right)-g_{d}\left(n\right)\right]\phi_{v}\left[u_{d}\left(m\right)-u_{d}\left(n\right)\right]$$

The surrogate function Q(U) is a convex approximate function of nonconvex optimization problem E(U). If a $U^{pre}$ with E is given, the following U can be obtained by solving Equation (29).

**Figure 3:**
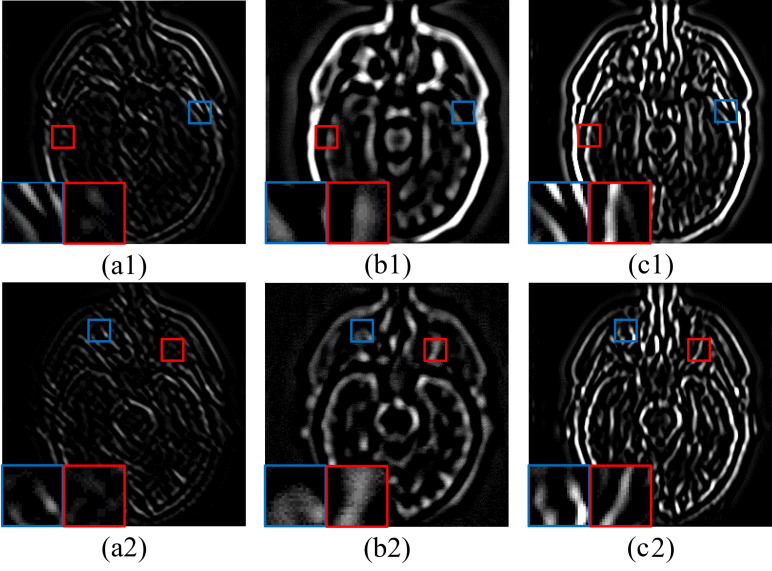
A comparison between the slices of high-frequency maps $U_{1,2}$ (**a1**,**a2**), original mixed-frequency maps $G_{a,b}$ (**b1**,**b2**), and complete mixed-frequency maps $U_{a,b}$ (**c1**,**c2**). The blue and red boxes are shown the enlarged details of each frequency map.

After the iteration, we obtain the complete mixed-frequency maps $U_{a,b}$. A comparison between high-frequency maps $U_{1,2}$(a1,2), original mixed-frequency maps $G_{a,b}$(b1,2), and completed mixed-frequency maps $U_{a,b}$(c1,2) can be seen in Figure 3. The blue frames show that $U_{a,b}$ completes the lost high-frequency information in $G_{a,b}$, while the red frames show that $U_{1,2}$ preserves and consolidates the product of mixed-frequency fusion. In a word, our JSDG method achieves the purpose of retaining both the structure of the input image and static guidance.

#### 4.4.2. Fusion of Complete Mixed-Frequency Maps

To retain as much detail as possible and reduce randomness and redundancy, we chose the components with a relatively high activity level in $U_{a/b}\in R^{I\times J\times D}$ to form our fused mixed-frequency map $H^{f}\in R^{I\times J\times D}$. For each coefficient, we used the activity fusion strategy in [29] with independent parameter settings. For notation simplicity, we use k(k∈a,b) to universally denote the source mix-frequency maps. Given the source sparse maps $U_{k}$, the corresponding initial activity level maps $M_{k}\in R^{I\times J}$ can be calculated as follows:

(30) $$\begin{array}{c} M_{k}\left(i,j\right)=\sum_{d=1}^{D}u_{k}\left(i,j,d\right),\left(1\leq i\leq I,1\leq j\leq J\right) \end{array}$$

The final activity level map can be calculated by taking the average process:

(31) $$\begin{array}{c} {M^{f}}_{k}\left(i,j\right)=\frac{\sum_{p=-r}^{r}\sum_{q=-r}^{r}M_{k}\left(i+p,j+q\right)}{{\left(2r+1\right)}^{2}},\left(1\leq i\leq I,1\leq j\leq J\right) \end{array}$$

where the parameter *r* is the window radius. Then, the fused mixed-frequency map $H^{f}$ can be calculated by:

(32) $$\begin{array}{c} H^{f}\left(i,j,:\right)=U_{k^{*}}\left(i,j,:\right),k^{*}=\underset{k}{\mathrm{argmax}}M_{k}^{f}\left(i,j\right),\left(1\leq i\leq I,1\leq j\leq J\right) \end{array}$$

### 4.5. Low-Frequency Completion

We processed the low-frequency maps $L_{1,2}$ by the WLE and WSEML method in [7] to obtain a fused low-frequency map $L^{f}\in^{I\times J}$. This is used as the base of the inverse nonsubsampled shearlet transform (INSST) in order to complete the lost low-frequency information. According to [7], because the amount of NSST decomposition is usually limited, low-frequency maps $L_{1,2}$ still contain some detailed information. Thus, to fully utilize the details from source images, we use the WLE method to fuse the low-frequency information and use the WSEML method to extract the remaining high-frequency information after the NSST. Thus, the fused low-frequency map $L^{f}$ can be calculated by the following equations:

(33) $$\begin{array}{c} L^{f}\left(i,j\right)=L_{n^{*}}\left(i,j\right),n^{*}=\underset{n}{\mathrm{argmax}}{\mathrm{WLE}}_{n}\left(i,j\right)\cdot {\mathrm{WSEML}}_{n}\left(i,j\right),\left(1\leq i\leq I,1\leq j\leq J\right) \end{array}$$

where

(34) $$\left\{\begin{array}{c} {\mathrm{WLE}}_{n}\left(i,j\right)=\sum_{p=-t}^{t}\sum_{q=-t}^{t}V\left(p+t+1,q+t+1\right)L_{n}\left(i+p,j+q\right)\\ {\mathrm{WSEML}}_{n}\left(i,j\right)=\sum_{p=-t}^{t}\sum_{q=-t}^{t}V\left(p+t+1,q+t+1\right){\mathrm{EML}}_{n}\left(i+p,j+q\right) \end{array}\right.$$

where V is a weighting matrix, *t* is the window radius, and EML means the Euclidean modified Laplacian.

### 4.6. Reconstruction Fused Image by the INSST

The INSST can reconstruct the fused image F in two steps, which are actually the inverse processes of multidirectional representation and multiscale decomposition in Section A. We use the fused mixed-frequency map $H^{f}\in R^{I\times J\times D}$ and fused low-frequency map $L^{f}\in R^{I\times J}$ as inputs. Firstly, the nonsubsampled pyramid $H^{f,k}\in R^{I\times J}\left(k=1,\ldots ,K\right)$ is generated by accumulating the filtered results in all directions. Secondly, the image is reconstructed from coarse to fine. The whole INSST can be represented by the following equation:

(35) $$\begin{array}{c} F=INSST\left(L^{f},H^{f}\right) \end{array}$$

## 5. Experiments

In this section, we first introduce the experimental settings, objective metrics, and comparison methods in detail. The proposed method is compared with other approaches in two aspects: a fused result analysis and a quantitative metrics analysis. To enrich the experimental content and improve the readability, we also carried out a parameter analysis, a convergence analysis, and an ablation analysis. All experiments were carried out using MATLAB R2016b on a computer with a dual-core Intel Core i5 processor (1.8 GHz) and 8GB 1600 MHz DDR3.

### 5.1. Experimental Settings

In this section, we discuss the experimental setup, including the images of the dataset used for the experiment, the selection of the comparison method, and the setting of the parameters. Other experimental details are shown in Table 2.

#### 5.1.1. Experimental Images

Medical image fusion technology widely uses four kinds of medical imaging, including computed tomography (CT), magnetic resonance imaging (MRI), positron emission tomography (PET), and single photon emission tomography (SPECT). In our experiment, the five most common multi-modal medical image fusion problems based on different modes were selected, including T1-T2, T2-PD and CT-MRI, MRI-PET, and MRI-SPECT. T1, T2, and PD are all MRI images based on different weights. The source images of our experiment were selected from the whole brain atlas database [30], and the spatial resolution was 256 × 256.

#### 5.1.2. Objective Metrics

To verify the performance superiority of our method, we selected a total of seven metrics to analyze the image fusion effect from different aspects. These metrics are divided into four categories in [29], namely information-theory-based metrics, image-feature-based metrics, image-structure-similarity-based metrics, and human-perception-inspired metrics. ChenBlum is a human visual system (HVS)-based metric [31] belonging to human-perception-inspired metrics, the purpose of which is to obtain the average value of the quality map of the whole image. Image-feature-based metrics include $Q_{abf}$ [32] and spatial frequency (SF) [33]. $Q_{abf}$ reflects the quality of the visual information obtained from the input image fusion, mainly the degree of edge information protection, and the SF measures the overall activity level of an image. The multi-scale structural similarity metric (MS-SSIM) [34] is an improved version of the SSIM [35]. Information-theory-based metrics include feature mutual information (FMI-pixel) and the nonlinear correlation coefficient (NCC) [36]. FMI-pixel calculates the mutual information of the image features and the NCC measures the general relationships among a group of images. The last one is the standard deviation (STD). It measures the contrast information of the fused image quality using variance.

#### 5.1.3. Comparison Methods

Our TDFusion model was compared with five existing medical image fusion methods, including ASR [37], NSST-PAPCNN [7], NSCT-PCDC [38], GFF [5], CS-MCA [29], and FCFusion [17]. Since CS-MCA is not designed for color image fusion, CA-MCA was not included in the comparison of color image fusion. In order to compare the fusion results of each method more objectively, all parameters in these methods are set to default values.

### 5.2. Visual Effects Analysis

Our comparative experiment was carried out on five medical image fusion problems, and three groups of results are selected for each problem. Then, we compared and analyzed the visual effects of various fusion methods in detail.

#### 5.2.1. Fusion Analysis on T1-T2

**Figure 4:**
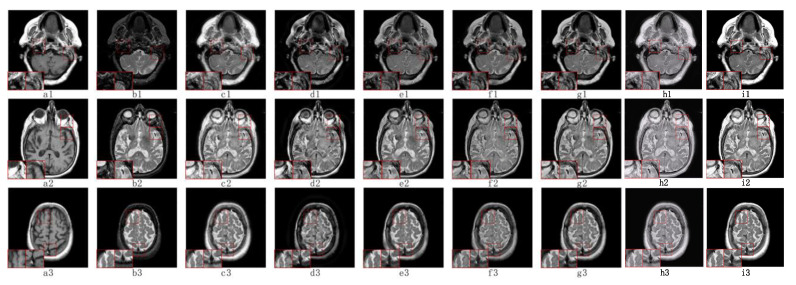
The visual effects of various fusion methods on T1 and T2 images. Each image has two close-ups. The first two columns of each group are the source images T1 (**a1**–**a3**) and T2 (**b1**–**b3**). The fused images are obtained by NSST-PAPCNN (**c1**–**c3**), NSCT-PCDC (**d1**–**d3**), GFF (**e1**–**e3**), ASR (**f1**–**f3**), CS-MCA (**g1**–**g3**), FCFusion (**h1**–**h3**), and TDFusion (**i1**–**i3**). The red boxes are shown the enlarged details of fused result.

The anatomical structure can be better observed in T1 images and T2 images are able to show tissue lesions more effectively. Figure 4(a1–a3) contains T1 images and T2 images are shown in Figure 4(b1–b3). The fusion of T1 and T2 images can lead to a more comprehensive anatomical structure and soft tissue information, which is of great significance in the clinical diagnosis and treatment of soft tissue lesions.

As shown in Figure 4, our TDFusion (i1–i3) model achieves the best visual effect of the fusion image, clearly shows the texture information of the original image, and excellently extracts the detailed information. ASR (f1–f3) and FCFusion (h1–h3) methods achieved good results in brightness and structure processing with the source image, but compared with our TDFusion model, the details are still not clear enough. CS-MCA (g1–g3) and GFF (e1–e3) methods lead to a lot of energy loss while extracting the corresponding details. In addition, NSCT-PCDC (d1–d3) and NSST-PAPCNN (c1–c3) methods not only have the problem of energy loss, but the detailed information extracted is also insufficient, resulting in the the non-ideal fusion image effect.

#### 5.2.2. Fusion Analysis on T2-PD

PD images mainly reflect the proton content of different tissues in the image; thus, the fusion with T2 images can better preserve the edge information and textural features of the source images and improve the efficiency of medical images for diagnosis.

**Figure 5:**
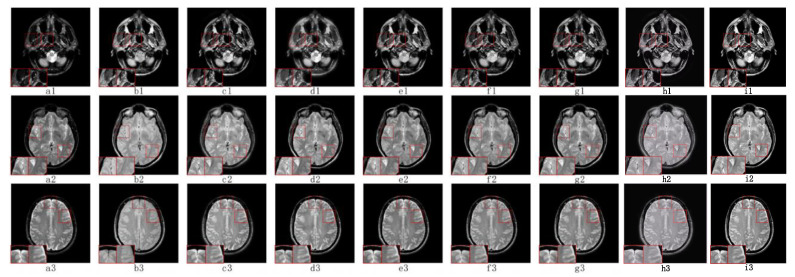
The visual effects of various fusion methods on T2 and PD images. Each image has two close-ups. The first two columns of each group are the source images T2 (**a1**–**a3**) and PD (**b1**–**b3**). The fused images were obtained by NSST-PAPCNN (**c1**–**c3**), NSCT-PCDC (**d1**–**d3**), GFF (**e1**–**e3**), ASR (**f1**–**f3**), CS-MCA (**g1**–**g3**), FCFusion (**h1**–**h3**), and TDFusion (**i1**–**i3**). The red boxes are shown the enlarged details of fused result.

As we can see in Figure 5, T2 source images and PD source images are shown in Figure 5(a1–a3,b1–b3), respectively. The fused images of NSST-PAPCNN (c1–c3) and NSCT-PCDC (d1–d3) are seriously interfered with by noise, many artifacts are produced, and the edges and details of the source image are not retained well, where c1 and d1 show these problems more obviously. This is not conducive to medical observation and research. Moreover, ASR (f1–f3) does not extract the information of the source image well, and the visual information quality of the fused image is not very good. In addition, the GFF (e1–e3) method suffers from information loss, the contrast of fusion image quality is poor, and the overall acquisition level is low. Although CS-MCA (g1–g2) and FCFusion (h1–h3) are relatively close to our TDFusion (i1–i3) fused image, our model has a better performance in terms of detail processing. In general, our method is very effective at removing noise and avoiding artifacts and achieves the best visual effect in all methods.

#### 5.2.3. Fusion Analysis on CT-MRI

Both MR images and CT images belong to anatomical imaging technology. CT images have a high-density spatial resolution, which can better reflect bone and other dense structures, while MRI can clearly reflect the soft tissue information. The fusion of MRI and CT images solves the problem of the poor representation of soft tissue lesions, which greatly improves the efficiency and accuracy of medical diagnosis.

**Figure 6:**
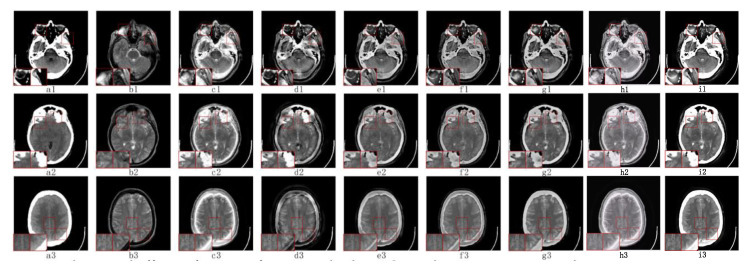
The visual effects of various fusion methods on CT and MR-T2 images. Each image has two close-ups. The first two columns of each group are the source images CT (**a1**–**a3**) and T2 (**b1**–**b3**). The fused images were obtained by NSST-PAPCNN (**c1**–**c3**), NSCT-PCDC (**d1**–**d3**), GFF (**e1**–**e3**), ASR (**f1**–**f3**), CS-MCA (**g1**–**g3**), FCFusion (**h1**–**h3**), and TDFusion (**i1**–**i3**). The red boxes are shown the enlarged details of fused result.

Figure 6 shows the fusion results of three sets of CT (a1–a3) and MR (b1–b3) images. As can be seen from Figure 6, NSCT-PCDC (d1-d3) and GFF (e1–e2) produce a large amount of energy loss, which greatly reduces the intensity and contrast of many fused images. The ASR (f1–f3) method overcomes most of the noise interference, but the problem of losing information still exists. In addition, NSST-PAPCNN (c1–c3) performs well in detail extraction and energy preservation, but it is not as good as CS-MCA (g1–g3) and TDFusion (i1–i3) at preserving texture information. Our TDFusion method has achieved good results in structure information extraction and contrast.

#### 5.2.4. Fusion Analysis on MRI-PET

PET belongs to functional imaging technology, which can well reflect the metabolic information of the human body, but the spatial resolution of functional images is often low. Therefore, the fusion of MR and PET can better combine anatomical and functional information, which is more conducive to medical observation and diagnosis. In addition, PET images provide information through color changes, so it is regarded as a color image which can be summarized by color fusion.

**Figure 7:**
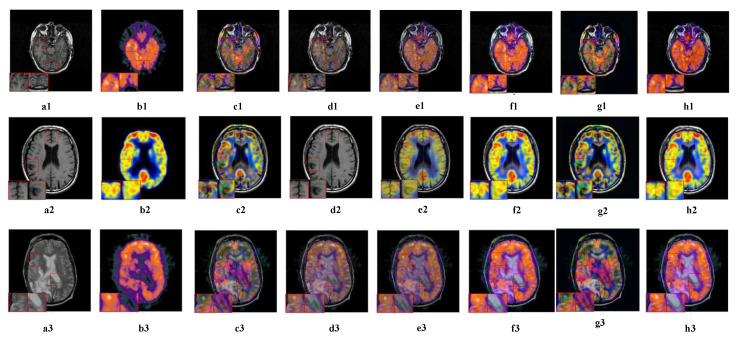
The visual effects of various fusion methods on MRI and PET images. Each image has two close-ups. The first two columns of each group are the source images MRI (**a1**–**a3**) and PET (**b1**–**b3**). The fused images are obtained by NSCT-PCDC (**c1**–**c3**), GFF (**d1**–**d3**), ASR (**e1**–**e3**), NSST-PAPCNN (**f1**–**f3**), FCFusion (**g1**–**g3**), and TDFusion (**h1**–**h3**). The red boxes are shown the enlarged details of fused result.

In Figure 7, the first two columns are the MR and PET source images. NSCT-PCDC and GFF suffer from severe color distortion, resulting in a low color fidelity. In Figure 7e2, no color information is extracted. The structural and textural information provided by ASR is not sufficient for final fused results, leading to much information loss and resulting in brightness. In addition, NSST-PAPCNN and FCFusion methods handle the color information well and achieve a higher visual quality than other methods, but they are still insufficient compared with TDFusion. Although our TDFusion is not particularly good at retaining source image information, it has greatly improved the extraction of details and structural information.

#### 5.2.5. Fusion Analysis on MR-SPECT

Similar to PET images, SPECT produces a three-dimensional color medical image that can reveal metabolic changes without structural information. At the same time, the color image fusion strategy combined with MR tissue structure information is used to locate functional information.

**Figure 8:**
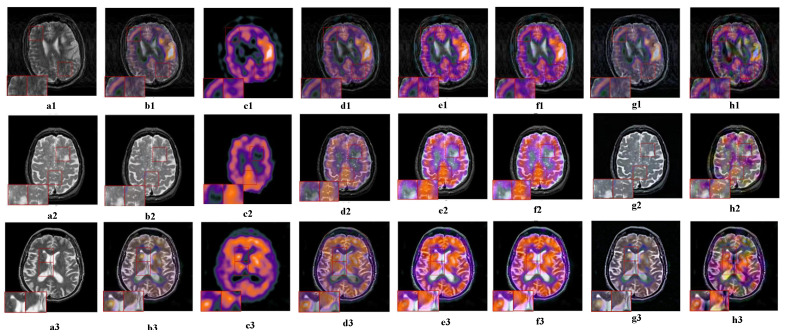
The visual effects of various fusion methods on MRI and SPECT images. Each image has two close-ups. The first two columns of each group are the source images MRI (**a1**–**a3**) and GFF (**b1**–**b3**). The fused images are obtained by PET (**c1**–**c3**), ASR (**d1**–**d3**), NSST-PAPCNN (**e1**–**e3**), NSCT-PCDC(**f1**–**f3**), FCFusion (**g1**–**g3**), and TDFusion (**h1**–**h3**). The red boxes are shown the enlarged details of fused result.

The three sets of fusion results are shown in Figure 8, and the visual effects of the fused images are also similar to MRI-PET fusion in the previous section. NSCT-PCDC, GFF, and ASR have different degrees of color distortion. Some important functional information contained in SPECT is lost, making medical diagnosis extremely difficult. In addition, FCFusion and our TDFusion have good structure and color information retention; however, NSST-PAPCNN is not as good as our model in edge information protection. The performance proves that our TDFusion has a good fusion effect on MRI and SPECT images.

### 5.3. Objective Metrics Analysis

In order to objectively analyze the performance of the fusion method, we randomly selected a total of 95 sets of image pairs, including 28 sets of T1–T2 image pairs, 23 sets of T2–PD image pairs, 12 sets of CT–MRI image pairs, 10 sets of MRI–PET image pairs, and 22 sets of MRI–SPECT image pairs. From Table 3, we can clearly see the performance results of the seven selected indicators for each fusion method. The top three results for each indicator are marked with red, blue, and green, respectively.

In grayscale fusion (T1–T2, T2–PD, CT–MRI), the performance of our TDFusion is the best in all indicators, whether this is in the extraction of details and structural information or in the processing of noise and energy loss. The similarity between the fusion images and the source images is very high, the visual effect is better, and the reliability of medical observations and diagnoses is improved.

In color fusion (MRI–PET and MRI–SPECT), ChenBlum, MS-SSIM, and SF are lower than other indexes, but remain in the top three. Regarding ChenBlum, the GFF method achieves the best results for the whole image quality, but at the same time, the color fidelity is low and the fused image is not ideal. MS-SSIM is an improvement of the SSIM method. The contrast is the maximum of all layers and the structure is related to all layers. The gap between our TDFusion and NSCT-PCDC is also very low; in addition, when SF is used as a measure of the overall level of activity, it is determined that our model results in a certain lack of information, which has been well improved by JSDG. From the previous part of the visual effect analysis, we can see that the image fused by our model has achieved good results in detail extraction, denoising, and color fidelity. The visual quality is also excellent. Although it has not reached the best results, it still remains the second or third best.

### 5.4. Analysis and Discussion

#### 5.4.1. Analysis of Computational Running Time

We further compare the computational efficiency of the task using the average running time presented in Table 4. As shown in Table 4, the proposed TDFusion achieves the best performance regarding the average running times, which demonstrates the superiority of the proposed TDFusion in efficiency.

#### 5.4.2. Convergence Analysis

**Figure 9:**
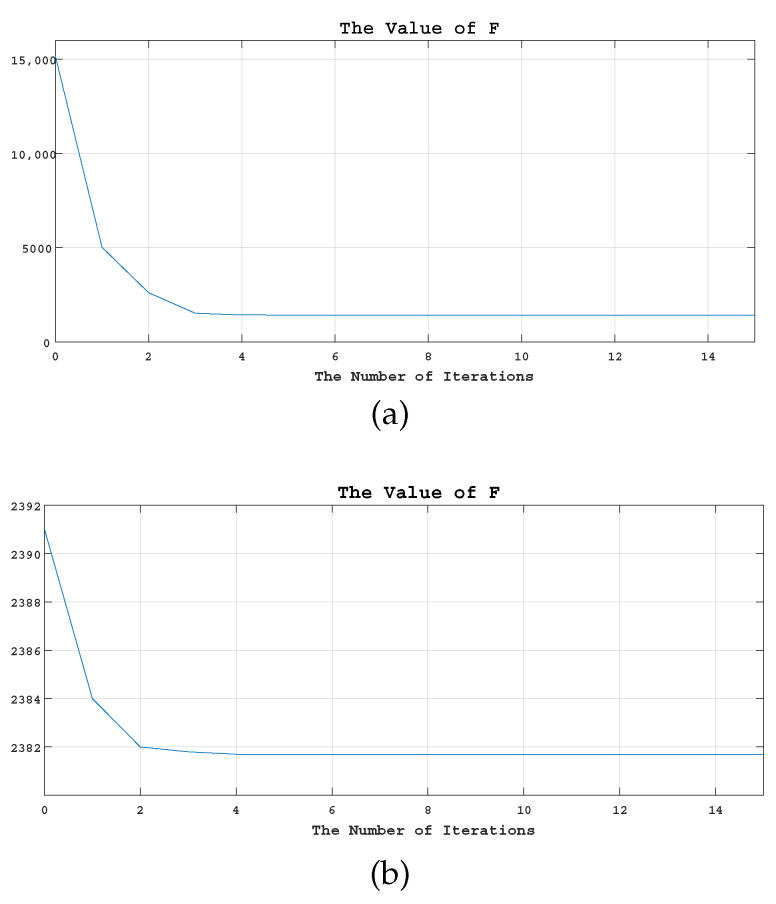
The convergence chain diagram of grayscale fusion and color fusion. From Equation (10), we present the converged value of *F* of grayscale fusion and color fusion in (**a**) and (**b**) respectively.

As can be seen from Figure 9a,b, which shows the cost fusion of TD and JSDG, respectively, our objective function converges after six iterations both in grayscale fusion or color fusion, indicating that our TDFusion model has good convergence.

#### 5.4.3. Ablation Analysis

**Figure 10:**
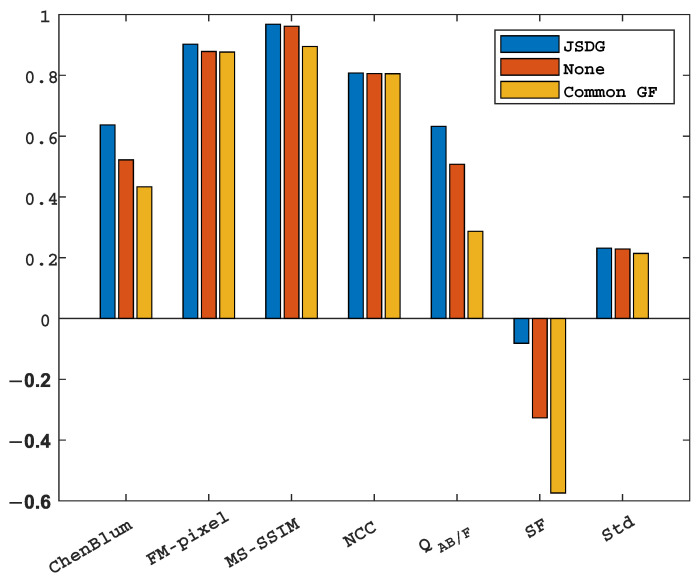
Ablation experimental data results. The indicators are ChenBlum, FM-pixel, MS-SSIM, NCC, $Q_{AB/F}$, SF, and STD.

A total of 91 groups of images (including five kinds of mode fusion) were selected. The results are shown in Figure 10. Common GF, JSDG, and None Filter were chosen for the ablation experiment as a comparison. The experimental results show that after supplementing the high-frequency information with JSDG, each index is significantly better than that without JSDG, but the performance of the universal filter is somewhat inferior to that of none filter in terms of index, which indicates that it cannot compensate for the high-frequency information of the image well.

#### 5.4.4. Parameter Analysis

There are two influential free parameters in our model, α and γ, which are the regularization parameter and fusion control parameter, respectively. They are related to the main cost functions of the two main optimization processes in our model: JSDG and TD. We used the control variable method to determine the parameter values, then tested the impacts of these values on the selected seven metrics. As can be seen from Table 5, except for $Q_{abf}$, the top three performances are obtained when γ = 1, which shows that it is reasonable to fix γ as 1. Then, we compared the results when γ is fixed to 1. It is easy to observe that the performance is similar in all indicators except $Q^{abf}$. Although the parameter combination of γ = 1 and α = 1 performs slightly better than other combinations of other indicators, the deterioration in $Q_{abf}$ is too significant, which leads to a decline in visual quality. Therefore, considering these data comprehensively, we set γ as 1 and α as 2. In the selection of parameters, we chose the average of 10 data images, not an individual image, and for the same values of α and γ, the impact on the overall results is not significant, so it does not lead to the problem of overfitting.

## 6. Conclusions

The non-downsampled shearlet transform domain tensor decomposition (TDFusion) approach for fusing medical images is proposed in this research. The source image is split into high-frequency and low-frequency components using the NSST method. The two components are fused independently using two different fusion techniques. The high-frequency and low-frequency parts are first combined using the TD method, and then the low-frequency part is subsequently combined using the WLE and WSEML methods in NSST-PAPCNN. Since HF and LF have different structural characteristics, some information will be lost during the fusion process. To lessen the negative impact of this information loss in the fusion process and enhance the fusion quality, we introduce JSDG. Finally, NSST reconstruction is used to obtain the final fusion results. The model exhibits notable advantages in detail and structural information extraction, energy preservation, and denoising according to a large number of successful experiments, including a parametric analysis, comparative experiments, and a visual effect analysis. In future research, we will consider using different methods to achieve low and high frequency separation to further optimize the model.

## Figures and Tables

**Table 1:** Symbols and meanings.

Symbols	Meanings
$a_{ijk}$	Scalar
*A*	Matrix
$A^{T}$	Conjugate transpose of a matrix
A	Third-order tensor
A (i,:,:)	Horizontal slice of the tensor A
A (:,j,:)	Side slices of tensor A
A (:,:,k)	The front slice of the tensor A
A (i,j,:)	Tube of Tensor A

**Table 2:** Experimental detailed specifications.

Experimental Environment	Parameters
Experimental equipments	Intel Core i5 dual-core processor 8GB 1600 MHz DDR3
Compiling software	MATLAB 2016b

**Table 3:** Objective performance of different fusion methods on seven metrics over T1–T2, T2–PD, CT–MRI, MRI–PET, and MRI–SPECT. The top three for each metric are marked in red, blue, and green.

Methods	ChenBlum	FMI-Pixel	MS-SSIM	NCC	$Q^{\mathrm{AB}/F}$	SF	Std
TDFusion	0.6514	0.8676	0.9667	0.8106	0.6605	−0.0776	0.3277
FCFusion	0.6260	0.8422	0.9146	0.8080	0.6390	−0.1547	0.2533
ASR	0.6370	0.8351	0.9261	0.8054	0.5960	−0.2086	0.2292
CS-MCA	0.6261	0.8430	0.9525	0.8058	0.6399	−0.1457	0.2550
GFF	0.6106	0.8477	0.9234	0.8067	0.6438	−0.1526	0.2606
NSCT-PCDC	0.5449	0.8275	0.8792	0.8042	0.5467	−0.1383	0.2427
NSST-PAPCNN	0.4459	0.8064	0.8679	0.8050	0.4031	−0.3536	0.2956
TDFusion	0.7622	0.8924	0.9813	0.8069	0.6450	−0.0882	0.2249
FCFusion	0.7486	0.8790	0.9622	0.8054	0.6330	−0.1447	0.1933
ASR	0.7606	0.8745	0.9669	0.8054	0.6167	−0.2392	0.1842
CS-MCA	0.7480	0.8821	0.9785	0.8055	0.6447	−0.1495	0.1976
GFF	0.7325	0.8793	0.9584	0.8056	0.6332	−0.1951	0.1881
NSCT-PCDC	0.6627	0.8674	0.9441	0.8046	0.5675	−0.1309	0.1838
NSST-PAPCNN	0.5787	0.8608	0.9520	0.8051	0.5434	−0.2473	0.2126
TDFusion	0.7122	0.9101	0.9328	0.8065	0.5957	−0.1014	0.3282
FCFusion	0.6860	0.8922	0.9146	0.8063	0.5390	−0.2547	0.2533
ASR	0.7114	0.9033	0.9081	0.8058	0.5468	−0.2691	0.2621
CS-MCA	0.6936	0.9080	0.9256	0.8059	0.5468	−0.2342	0.2887
GFF	0.6956	0.9054	0.8263	0.8061	0.5835	−0.2906	0.2529
NSCT-PCDC	0.6075	0.8961	0.8369	0.8052	0.5350	−0.1789	0.2695
NSST-PAPCNN	0.5449	0.8850	0.8911	0.8058	0.5168	−0.2833	0.3264
TDFusion	0.6398	0.8970	0.999994	0.8115	0.8188	−0.0148	0.2526
FCFusion	0.6365	0.8852	0.99994	0.8114	0.8090	−0.0127	0.2536
ASR	0.6386	0.8533	0.999947	0.8043	0.7545	−0.1419	0.1656
GFF	0.6569	0.8928	0.999996	0.8112	0.8136	−0.0263	0.2427
NSCT-PCDC	0.5970	0.8952	0.999996	0.8106	0.8146	−0.0136	0.2470
NSST-PAPCNN	0.6438	0.8958	0.999995	0.8113	0.7811	−0.0120	0.2524
TDFusion	0.6588	0.8972	0.999983	0.8094	0.7719	−0.0302	0.2627
FCFusion	0.6510	0.8962	0.999980	0.8086	0.7310	−0.0547	0.2413
ASR	0.6511	0.8655	0.999955	0.8049	0.6826	−0.1884	0.1803
GFF	0.6845	0.8959	0.999986	0.8091	0.7682	−0.0469	0.2438
NSCT-PCDC	0.6261	0.8967	0.999992	0.8084	0.7315	−0.0310	0.2521
NSST-PAPCNN	0.6454	0.8960	0.999987	0.8088	0.7215	−0.0278	0.2618

**Table 4:** The average running time of different methods.

Methods	CS-MCA	GFF	NSCT-RPCNN	NSCT-PCDC	NSST-PAPCNN	FCFusion	TDFusion
Times	137.38	0.06	8.43	15.14	6.86	56.89	20.66

**Table 5:** The comparison of metric values with different parameter values.

	ChenBlum	FMI-Pixel	MS-SSIM	NCC	$Q^{\mathrm{AB}/F}$	SF	std
γ=1,α=1	0.6167	0.89140	0.965741	0.80886	0.5990	−0.0855	0.25175
γ=1,α=2	0.6137	0.89136	0.965740	0.80877	0.6033	−0.0865	0.25174
γ=1,α=3	0.6117	0.89124	0.965731	0.80874	0.6065	−0.0871	0.25173
γ=10,α=1	0.6054	0.89081	0.965602	0.80850	0.6295	−0.0911	0.25164
γ=10,α=2	0.5981	0.89045	0.965476	0.80815	0.6309	−0.0978	0.25149
γ=10,α=3	0.5927	0.89007	0.965344	0.80793	0.6321	−0.1043	0.25132
γ=0.1,α=1	0.5919	0.89053	0.965139	0.80802	0.6145	−0.0941	0.25155
γ=0.01,α=1	0.5867	0.89038	0.964926	0.80791	0.6115	−0.0957	0.25150
γ=0.1,α=2	0.5788	0.89026	0.964749	0.80778	0.6052	−0.0980	0.25142
γ=0.01,α=2	0.5704	0.89008	0.964429	0.80766	0.6022	−0.1003	0.25132
γ=0.1,α=3	0.5702	0.89006	0.964499	0.80765	0.6009	−0.1008	0.25130
γ=0.01,α=3	0.5603	0.88983	0.964045	0.80752	0.5985	−0.1036	0.25117

## Data Availability

Not applicable.
